# Anti-Amyloidogenic Properties of Some Phenolic Compounds

**DOI:** 10.3390/biom5020505

**Published:** 2015-04-17

**Authors:** Afsaneh Porzoor, Benjamin Alford, Helmut M. Hügel, Danilla Grando, Joanne Caine, Ian Macreadie

**Affiliations:** 1 School of Applied Sciences, RMIT University, Bundoora, Victoria 3083, Australia; E-Mails: afsaneh.porzoor@rmit.edu.au (A.P.); danilla.grando@rmit.edu.au (D.G.); 2 School of Applied Sciences, RMIT University, Melbourne, Victoria 3000, Australia; E-Mails: ben.alford@amptechpl.com (B.A.); helmut.hugel@rmit.edu.au (H.M.H.); 3 Materials Science and Engineering, CSIRO Preventative Health Flagship, 343 Royal Parade, Parkville, Victoria 3052, Australia; E-Mail: Jo.Caine@csiro.au

**Keywords:** Alzheimer’s disease, *Saccharomyces cerevisiae*, polyphenols, *AHP1*, anti-amyloidogenic, danshen

## Abstract

A family of 21 polyphenolic compounds consisting of those found naturally in danshen and their analogues were synthesized and subsequently screened for their anti-amyloidogenic activity against the amyloid beta peptide (Aβ_42_) of Alzheimer’s disease. After 24 h incubation with Aβ_42_, five compounds reduced thioflavin T (ThT) fluorescence, indicative of their anti-amyloidogenic propensity (*p* < 0.001). TEM and immunoblotting analysis also showed that selected compounds were capable of hindering fibril formation even after prolonged incubations. These compounds were also capable of rescuing the yeast cells from toxic changes induced by the chemically synthesized Aβ_42_. In a second assay, a *Saccharomyces cerevisiae AHP1* deletant strain transformed with GFP fused to Aβ_42_ was treated with these compounds and analyzed by flow cytometry. There was a significant reduction in the green fluorescence intensity associated with 14 compounds. We interpret this result to mean that the compounds had an anti-amyloid-aggregation propensity in the yeast and GFP-Aβ_42_ was removed by proteolysis. The position and not the number of hydroxyl groups on the aromatic ring was found to be the most important determinant for the anti-amyloidogenic properties.

## 1. Introduction

Alzheimer’s disease (AD) has been classified as one of the protein misfolding diseases due to the presence of amyloidogenic protein, in particular amyloid beta (Aβ_42_) and its tendency to misfold and aggregate into insoluble, toxic amyloid fibrils. Various sizes of Aβ oligomer have been associated with the onset and progression of AD [1,2]. For example, oligomeric forms have been shown to induce synaptic dysfunction [3] and SDS-stable oligomers were found to be neurotoxic [4], whereas Aβ monomers and mature fibrils are relatively inert [5,6]. Therefore, targeting the Aβ oligomerization and aggregation pathway has become a key focus for discovering therapeutics that may be capable of preventing or delaying the onset of AD [7]. Studies have shown that the anti-parallel β-sheet orientation of the peptide, and hence the aggregation, could be blocked by compounds that can act as β-sheet breakers or ligands [8,9].

There is a growing consensus in the literature that the frequently investigated polyphenols could be key molecules for the development of therapeutics or as a part of a prevention strategy for AD [10,11,12,13,14]. Polyphenols are present widely in fruits and vegetables. The chemo-protective effects of (−)-epigallocatechin-3-gallate (EGCG), a polyphenol in green tea, as an anti-aging and cancer prevention agent have been well documented [15,16,17,18,19,20,21]. It has been proposed that oxidized EGCG molecules prevent toxicity and dissociation of Aβ peptide by covalent cross-linking and formation of Schiff bases with free amine groups/residues within the fibrils [22]. Although epidemiological studies have shown that consumption of red wine, coffee, rosemary, curcumin, and many other polyphenols can have a protective effect against Alzheimer’s disease and even reverse the cognitive impairment [23], only few studies have investigated the effect of small molecules on the Aβ_42_ aggregation and toxicity in a simpler but powerful model like yeast. This model previously has been proven to be important for its contribution in identification of β-secretase activity [24] and determining the necessary component for biological activity and function of γ-secretase [25], as well as autophagic response to Aβ_42_ toxicity [26]. Another microbial model reported by Perez *et al.* utilizes *Escherichia coli* to study the inhibition of amyloid production by Aβ_42_ and tau: this microbe has also been employed in polyphenolic compounds [27].

The Chinese herbal medicine danshen (*Salvia miltiorrhiza*) has been associated with many health benefits. Recent analysis has shown that the presence of both lipid- and water-soluble active phytochemical constituents in danshen can inhibit the formation of Aβ_42_ fibrils (reviewed in [28]). The largest group of plant phenolics in the diet are the hydroxycinnamic acids (HCA), namely caffeic acid, ferulic acid, and chlorogenic acid (reviewed in [29]). Natural polyphenols that are regularly consumed in foods have shown promising therapeutic effects and attracted great research attention [30]. Danshen constituents have also been shown to possess potential therapeutic effects in the treatment of AD in both animal models by attenuating cognitive dysfunction induced by the Aβ_42_ peptide [31,32,33] and in tissue culture studies [34,35,36,37] due to protection against the toxicity of the amyloid peptide.

In this work a group of 21 phenolic compounds related to those from danshen and their analogues (mainly hydroxybenzoic acid (HBA) derivatives) were screened for activity against the oligomerization of Aβ_42_. Since the number and position of the hydroxyl moieties attached to the phenyl rings in benzoic acid and cinnamic acid analogs are variable, this allowed the investigation of the role the hydroxyl groups play in the amyloid inhibitory activity of these phenolic acids. Further, in order to explore whether the hydroxyl group possess unique or essential antiamyloidogenic activity in those phenolic acids, various methoxy benzoic and cinnamic acid derivatives were also examined.

## 2. Results and Discussion

### 2.1. Inhibitory Effects of Phenolic Compounds on Aβ_42_ Aggregation

The ability of phenolic compounds to inhibit the formation of Aβ_42_ oligomers and aggregates was monitored by co-incubating freshly prepared Aβ_42_ with phenolic compounds and measuring the Thioflavin T (ThT)-induced fluorescent intensity. Because the colors associated with many of these compounds might interfere with ThT, modulate the fluorescence yield of ThT, and bias the results [38], analyses of these compounds without addition of Aβ_42_ peptide were also included. This allowed adjustment of the baselines and elimination of the effect of color on the photometric readings or quenching of the ThT fluorescence in this assay.

**Figure 1:**
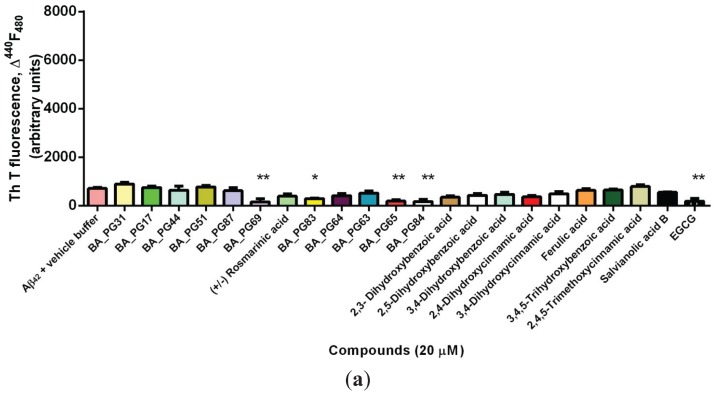

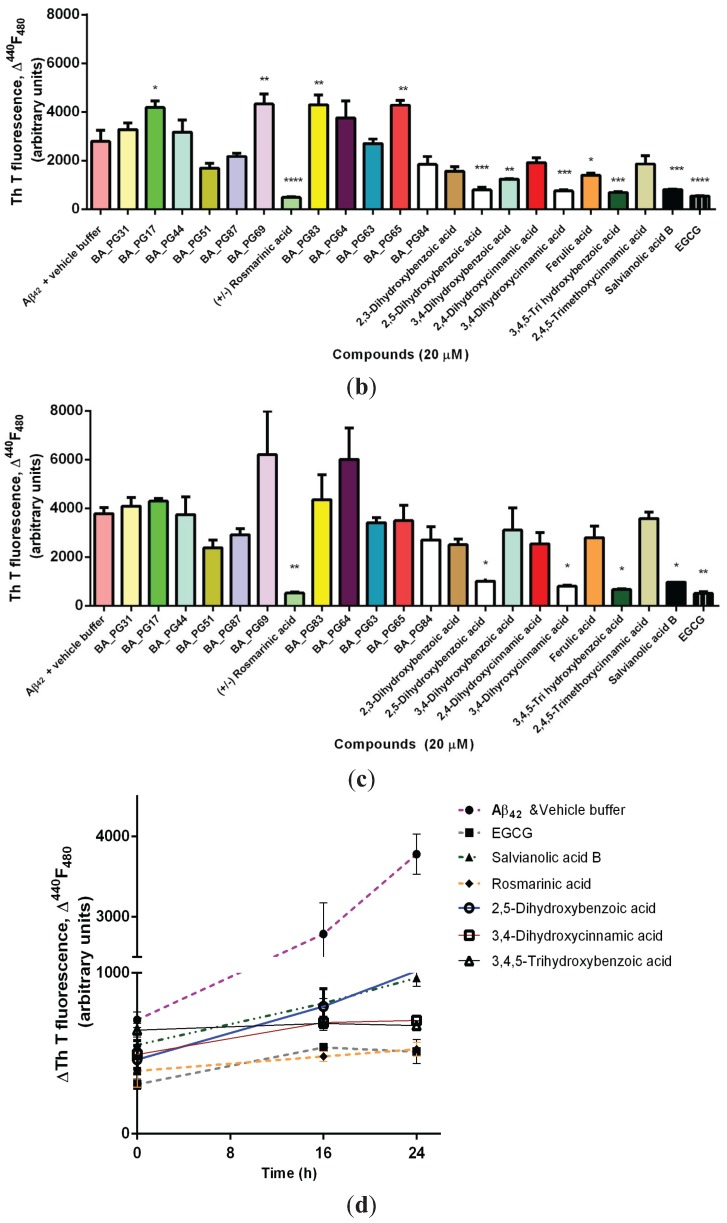
Amyloid formation assayed by thioflavin T fluorescence. (**a**) Fibril formation of Aβ_42_ and the associated increase in *in situ* ThT fluorescence in presence and absence of compounds were measured at 0 h (baseline) (**b**) after 16 h (**c**) and after 24 h incubation; (**d**) Selected phenolic compounds that resulted in a significant anti-amyloidogenic property compared with the positive control (EGCG) and negative control (Aβ_42_ & vehicle buffer) determined by ThT measured at 0, 16, and 24 h after incubation with chemically synthesized Aβ_42_. Significant differences are indicated: * *p* < 0.05; ** *p* < 0.01; *** *p* < 0.005; **** *p* < 0.001.

At the baseline (0 h) the starting fluorescent intensity was significantly lower in a positive control (EGCG) compared with Aβ_42_ and vehicle buffer (*p* < 0.01; Figure 1a). A similar effect was also viewed in the presence of caffeic acid trimers (BA_PG65, BA_PG69), chalcone (BA_PG84), and—to lesser extent—the Benzo[*b*]furan derivative (BA_PG83) (*p* < 0.05). Benzofuran-based compounds have previously been shown to exhibit potential aggregation and neurotoxicity inhibition effect against Aβ_42_ [39,40].

After 16 h incubation, ferulic acid and 3,4-DHBA reduced the ThT, but this effect was transient and was not observed after 24 h incubation (Figure 1b). Ferulic acid has been shown [41] to disrupt the Aβ_42_ preformed fibrils through aromatic hydrophobic interactions and hydrogen bonding with the amyloid peptide.

**Figure 2:**
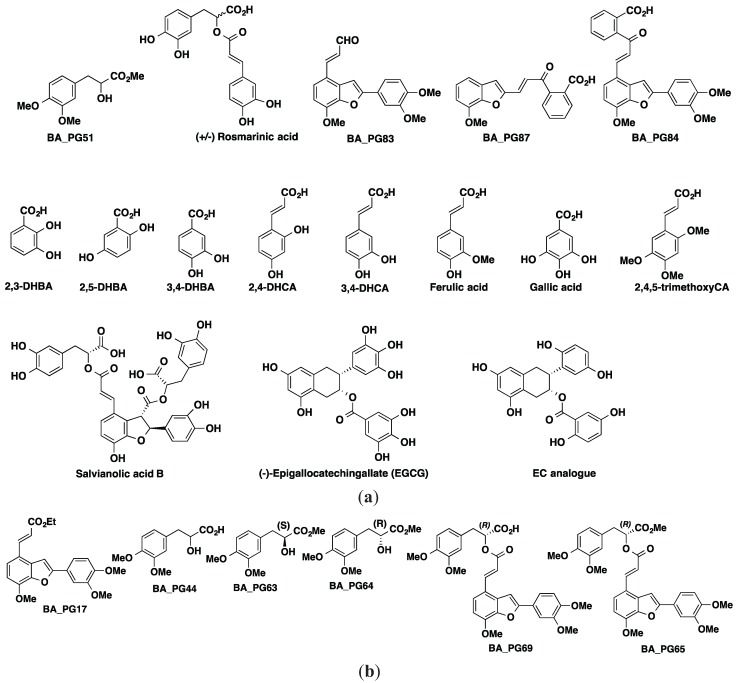
Chemical structures of analyzed polyphenolic compounds. (**a**) Compounds that reduced ThT associated fluorescent similar to EGCG; (**b**) compounds that were not found to be potent inhibitors of amyloid aggregation.

After 24 h co-incubation of compounds with Aβ_42_, rosmarinic acid, similar to EGCG (*p* < 0.001), exerted the most potent inhibitory effect on peptide aggregate formation (Figure 1c). Other compounds that showed aggregation inhibition capacity were 2,5-DHBA (gentisic acid), 3,4-DHCA (caffeic acid), 3,4,5-THBA (gallic acid), and salvianolic acid B (*p* < 0.005). 3,4-DHBA also significantly reduced the aggregation propensity of Aβ_42_ compared with the control sample (*p* < 0.01). On the other hand, caffeic acid trimers BA_PG65 and BA_PG69, as well as compounds BA_PG83 (*p* < 0.01) and BA_PG17 (*p* < 0.05), significantly contributed to the increased ThT associated fluorescence after 24 h co-incubation with chemically synthesized Aβ_42_. Further, the results suggest that these compounds accelerated the aggregation and fibril formation capacity of the peptide compared with the control sample (Figure 2a). Five compounds lowered the fluorescent intensity, indicating their capability in slowing aggregation and fibril formation (ThT negative). These were rosmarinic acid, 2,5-DHBA, 3,4-DHCA, 3,4,5-THBA (gallic acid), and salvianolic acid B (*p* < 0.05) (Figure 1d). The kinetic effect of EGCG on Aβ_42_ fibrillogenesis inhibition was unlike that of the other compounds. EGCG interacts early with Aβ_42_ and dissociates oligomer formation in a sustainable manner.

The therapeutic effect of many polyphenols has been attributed to the presence of aromatic rings, namely phenyl rings and hydroxyl groups, which result in decreased fibril formation [42]. The effect of these aromatic residues is attributed to their hydrophobic interactions in the π-stacking interaction with aromatic amino acids in the amyloid, and halting the β-amyloid self-assembly process that leads to oligomerization [43,44]. Ono *et al.* [45] have indicated that the greater the number of hydroxyl groups present, the higher the anti-amyloidogenic activity. Our results show that the number of hydroxyl moieties attached to the phenyl ring is not the major determinant. After analyzing the positional isomers, it became clear that the position of the hydroxyl moieties on the aromatic ring is the main reason for their inhibitory potency. Overall, the effect of these aromatic residues in polyphenols is due to their ability to prevent transition of α-helix conformation to β-sheet and therefore fibril formation [46]. Also, it became apparent that the positions of the hydroxyl groups may be more important than their numbers in the structure for their optimum biological activity. Among the compounds tested, simple benzoic acid derivatives identified by the number and positions of hydroxyls on the aromatic ring displayed different anti-oligomerization activities. At the baseline both 3,4-DHBA and 2,5-DHBA isomers [47] (refer to Figure 2 for chemical structures) were active in hindering oligomer and fibril formation, and after 24 h incubation only 2,5-DHBA remained inhibitory. Therefore, the position of hydroxyl groups on the 2,5-DHBA isomer contributed to the anti-oligomerization potency compared with other DHBA isomers and trihydroxybenzoic acid (THBA) (Figure 2a). 2,5-DHBA, however is known not to detoxify existing amyloid fibrils [47]. 3,4,5-THBA (gallic acid), unlike 2,3, 2,5-, and 3,4-DHBA, was reported by LeVine *et al.* [47] to stabilize biotinyl-Aβ_42_ oligomers and block their dissociations in the presence of active dissociators.

The time comparisons of the data in Figure 1b,c suggests that the greater lipophilicity of the methoxy ether and ester derivatives BA-PG69 and BA-PG65 of (+/−) rosmarinic acid did not enhance Aβ_42_ aggregation in the yeast model. The 24 h incubation of 2,4,5-trimethoxycinnamic acid (THCA) showed a similar lack of potency with time. This work therefore raises the question whether the hydrogen bonding nature and the position of the hydroxyl groups, shown in the EC analogue structure in Figure 2a, may exhibit potent anti-amyloidogenic properties. Replacing −OH groups with methoxy on inositol has been shown to stabilize the protofibrils *in vitro* and attenuate the spatial memory impairments in mice model of AD [48]. Other compounds—BA_PG44, BA_PG51, BA_PG87, BA_PG63, and BA_PG64—did not show similar potency to EGCG (Figure 2b). EGCG has been shown to redirect amyloidogenic peptide into unstructured, non-toxic and off-pathway oligomers [49].

**Figure 3:**
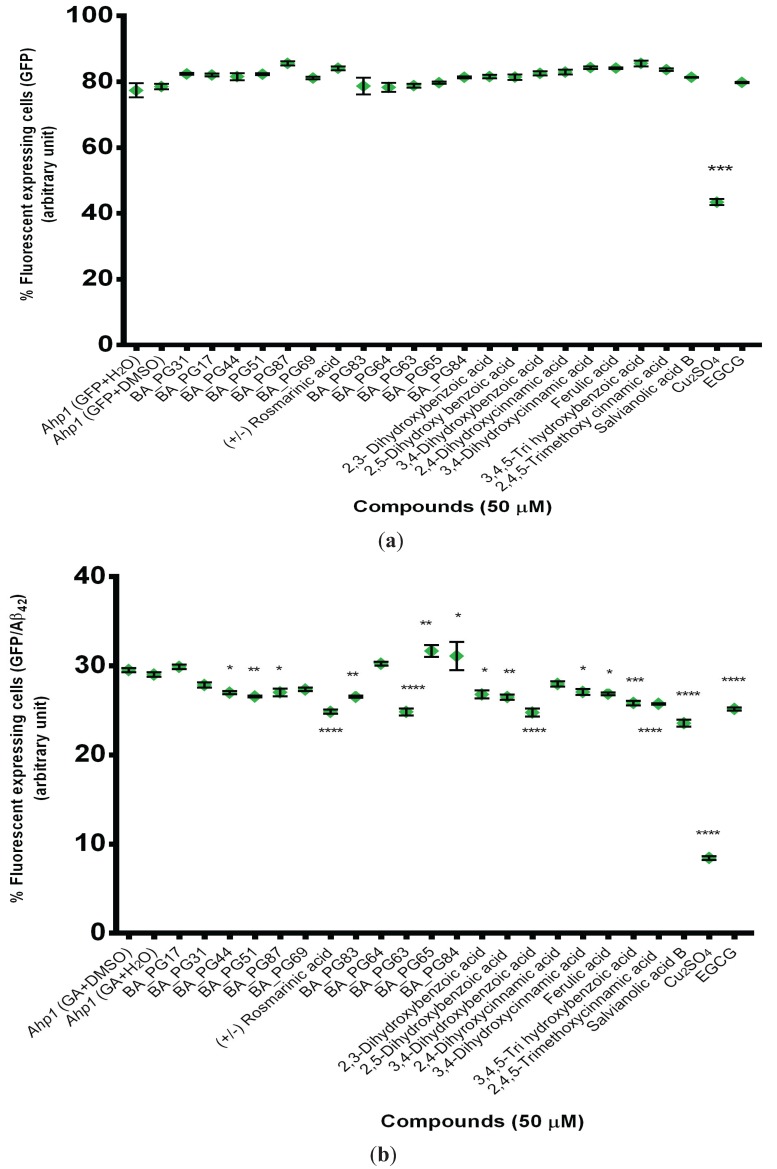
(**a**) Flow cytometry analysis of transformants of *Ahp1* strain with GFP and (**b**) GFP-Aβ_42_ in the presence of polyphenolic compounds.

High throughput *in vivo* screening of compounds that inhibit aggregation of Aβ_42_ using green fluorescent protein (GFP) fusion has been reported previously [50,51,52]. Therefore, compounds were screened for their effect on the reduction or increase in fluorescence due to aggregation of Aβ_42_ in *AHP1* deletant strain *S. cerevisiae* transformants expressing GFP-Aβ_42_. The alkyl hydroperoxide reductase protein (Ahp1p) is a thiol-specific peroxiredoxin that provides protection against reactive oxygen species (ROS) [53,54]. Using this mutant strain *Ahp1* should allow identification of those compounds that can provide protection against oxidation and those capable of inhibition of amyloid aggregation in the GFP-Aβ_42_ fusion-associated fluorescence assay. This screening is based on the percentage of fluorescence emission from the correct folding of GFP as a reporter for Aβ_42_ aggregation in the presence of compounds. The GFP fusion system used in this study has been described previously [51]. This GFP-Aβ_42_ fusion results in the green fluorescent being visible in punctuate patches. In the GFP expression alone, the GFP is distributed uniformly throughout the cell. The green fluorescence associated with GFP alone was very strong and present in most cells compared with the GFP-Aβ_42_ fusion. In fact, in vehicle buffer (DMSO) control samples the GFP florescence was observed in ~80% of cells (78.5 ± 1.4) (Figure 3a). This level did not change significantly in the presence of 50 μM phenolic compounds. The selected ThT negative compounds also reduced the fluorescence associated with the GFP-Aβ_42_ fusion (Figure 3b).

Fourteen compounds reduced the green fluorescence associated with GFP-Aβ_42_ fusion (Figure 3b). Reduction of green fluorescence has been reported to be the result of autophagy and proteolysis response [55]. Rosmarinic acid, salvianolic acid B, BA_PG63, 2,4,5-trimethoxyCA, 3,4,5-THBA, and 3,4-DHBA reduced the fluorescence level similar to EGCG (*p* < 0.001). Compounds such as BA_PG65 (*p* < 0.01) and BA_PG84 (*p* < 0.05) increased the green fluorescence associated with GFP-Aβ_42_ fusion and also showed immediate anti-oligomerization properties in the ThT assay (Figure 1a). Other compounds such as BA_PG44, BA_ PG87, BA_PG51, and BA_PG63 did not show high anti-oligomeric potency when tested by the ThT assay (Figure 1a), whereas they significantly decreased the green fluorescence associated with GFP-Aβ_42_ fusion in the yeast model (Figure 3b). This further indicates that the effect of these compounds on Aβ_42_ aggregation could be different in *in vivo* biological systems relative to the *in vitro* systems due to interactions with other cellular components and mechanisms.

### 2.2. Protection Effect of Selected Inhibitors against the Cytotoxicity of Aβ_42_

**Figure 4:**
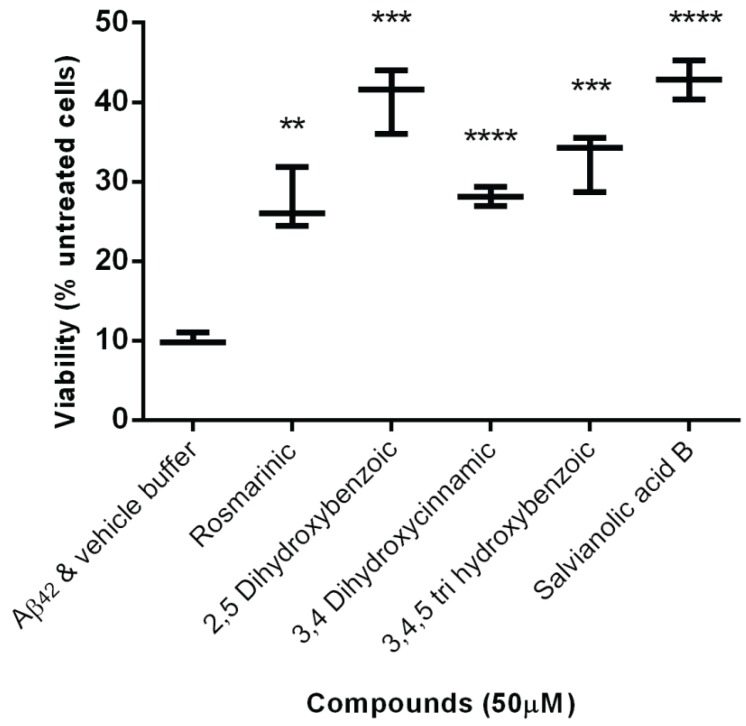
Cytotoxicity effect of freshly prepared HFIP pretreated Aβ_42_ peptide (5 μM) on *C. glabrata* cells.

The cytotoxicity effect of 5 μM hexafluoroisopropanol (HFIP) pretreated Aβ_42_ [56] and the protection of the five selected phenolic compounds (50 μM) were tested on *C. glabrata* cells. The survival rate of yeast cells in the presence of Aβ_42_ was only ~10% of the untreated control sample (Figure 4). All of the selected phenolic compounds were capable of inhibiting toxicity to the *C. glabrata* cells when compared with the control. Salvianolic acid B (*p* < 0.001), gentisic acid, gallic acid, and caffeic acid (*p* < 0.005) were the most effective (Figure 3).

It should be noted that a synthetic Aβ_42_ peptide was utilized for the cytotoxicity assay and according to previous reports, the oligomers formed by synthetic Aβ_42_ are less stable than naturally occurring oligomers [57]. Endogenous Aβ has also been shown to be more potent than synthetic Aβ_42_ [58]. Despite these dissimilarities, synthetic Aβ_42_ peptide is used widely for the identification of anti-amyloidogenic compounds [59,60,61,62]. It should therefore be expected that the biological potency of selected chemicals on inhibiting the aggregation of Aβ_42_ oligomers may not be similar under different conditions. The mechanisms by which the selected compounds rescued cells from Aβ_42_ oligomer toxicity could be explained by two distinct pathways. Firstly, the compounds may not be cell-permeable and are attaching to the cell wall and forming a bond with the surface of the yeast, thereby limiting Aβ_42_ contact and association and secondly, the compounds generate nontoxic Aβ_42_ forms with no cellular toxicity.

### 2.3. Selected Compounds Do not Inhibit Aβ_42_ Oligomer Formation (TEM)

To further explore the effects of the selected ThT negative phenolic compounds (Figure 1c) on amyloid aggregation and fibril formation, morphological changes during 7 day incubation of Aβ_42_ peptide in the presence of these five selected compounds were examined by TEM. A control sample of chemically synthesized Aβ_42_ peptide with only the addition of vehicle buffer was analyzed for morphological comparison.

**Figure 5:**
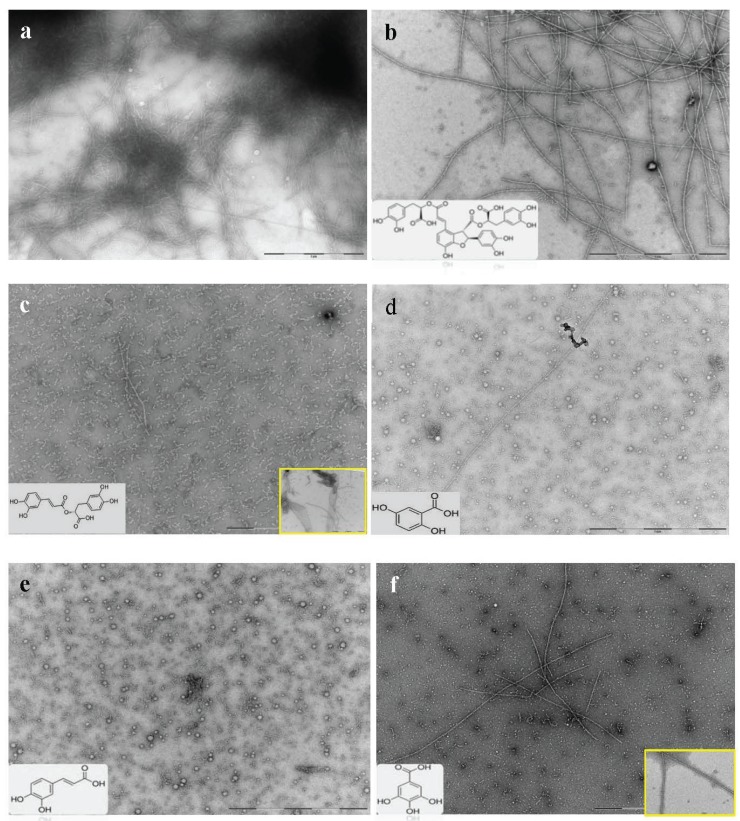
TEM micrograph of Aβ_42_ co-incubated with selected compounds. (**a**) Morphological effects of selected compounds on chemically synthesized Aβ_42_ aggregation was investigated and compared with the control sample. (**b**) Micrograph of chemically synthesized Aβ_42_ in presence of salvianolic acid B; (**c**) rosmarinic acid; (**d**) 2,5-DHBA (gentisic acid); (**e**) 3,4-DHCA (caffeic acid); and (**f**) 3,4,5-THBA (gallic acid). Arrows show micelle (globular) structures, protofibrils, and fibrillar species of Aβ_42_. A micrograph showing other conformational species of peptide formed by the compound is highlighted in the yellow box. The structure of each compound is also shown on the micrograph. (Scale bars = 200 nm).

The control Aβ_42_ sample resulted in highly ordered fibrillar structures (Figure 5a). Although none of the compounds completely prevented aggregation, the extent of protofibril and fibril formation varied in the presence of different compounds. Structures like sheets, globular, and short or long fibrils were formed as a result of the affect of the compounds on the Aβ_42_ conformation.

Salvianolic acid B resulted in the formation of high molecular weight (HMW) species including protofibrils and mature amyloid fibrils. The fibrils were well separated and dispersed compared with the control sample (Figure 5b). Rosmarinic acid, on the other hand, presented a potency of aggregation inhibition but prolonged incubation resulted in formation of oligomers, protofibrils, long fibrils, and clusters of short fibrillar structures that were merged into sheet-like structures (Figure 5c). In the presence of 2,5-DHBA (gentisic acid) (Figure 5d), micelle-like structures [63] along with many protofibrils became visible. However, few fibrillar structures that were very long in length were detected. Caffeic acid also formed spherical micelle-like species (Figure 5e). The globule structures presumably represented caffeic acid microdroplets in which Aβ_42_ had deposited and begun to aggregate. After 7 days’ co-incubation, caffeic acid proved to be a good ligand for the Aβ_42_ peptide, preventing extensive aggregation and fibril formation. 3,4,5-THBA (gallic acid; Figure 5f) resulted in the formation of only a few very long, twisted fibrils. Both 3,4-DHCA and 3,4,5-THBA resulted in the formation of amorphous and spherical structures that are known to be non-toxic, although the exact molecular mechanism underpinning their inhibitory effect in not clear at this point. It indicated, however, that a direct binding of these molecules with intrinsically disordered Aβ_42_ promotes their assembly.

One possible explanation for the detection of a high level of fibrillar structures in the presence of salvianolic acid B and rosmarinic is that these two compounds are capable of accelerating fibril formation after prolonged incubation, resulting in lesser amounts of toxic soluble oligomer formation. Another possible explanation is that these selected ThT negative compounds competitively bind to the β-sheet site of the amyloid fibrils resulting in the inhibition or a reduction in the ThT interactions with these active sites along the length of the fibrils, causing a lower fluorescent emission associated with ThT [38].

### 2.4. PAGE and Western-Blot of Aβ_42_ Co-Incubated with Selected Potent Compounds

The selected phenolic compounds were investigated for their capacity to prevent oligomeric Aβ_42_ formation after prolonged incubation. SDS-PAGE of the Aβ_42_ conformers that formed over 7 days; incubation in the presence of ThT negative compounds were analyzed by silver staining and Western blot using WO2 monoclonal antibody against the Aβ peptide.

**Figure 6:**
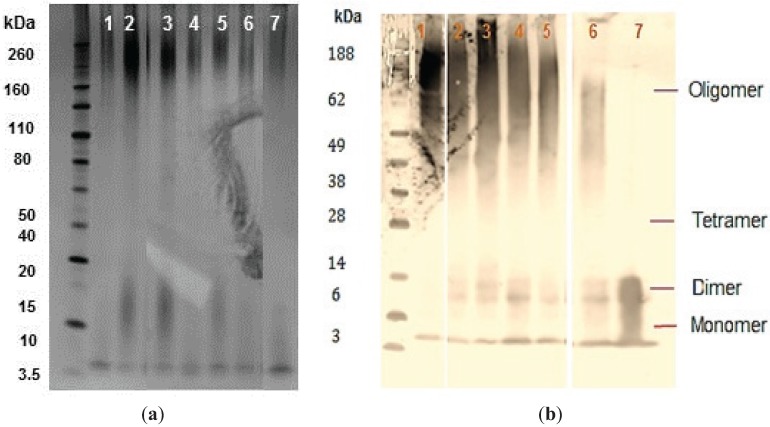
SDS-PAGE analyses of Aβ_42_ conformers in the presence and absence of compounds. (**a**) Peptide solutions co-incubated with the selected phenolic compounds were fractioned by SDS-PAGE electrophoresis (4%–12% Bis-Tris gel). One hundred nanograms of each Aβ_42_ sample were analyzed by silver staining. Samples are vehicle buffer control and Aβ_42_ (lane 1), salvianolic acid B (lane 2), rosmarinic acid (lane 3), 2,5-DHBA (lane 4), 3,4-DHCA (lane 5), 3,4,5-THBA (lane 6), and freshly prepared Aβ_42_ (lane 7). Lane M contains molecular weight marker. Half a microgram of each Aβ_42_ sample (from TEM experiment) was also analyzed by immunoblotting (Section 2.4) using anti-Aβ WO2 antibody; (**b**) 0.5 μg of each Aβ_42_ sample (from TEM experiment) was also analyzed by immunoblotting using anti-Aβ WO2 antibody. Samples are salvianolic acid B (lane 1), rosmarinic acid (lane 2), 2,5-DHBA (lane 3), 3,4-DHCA (lane 4), 3,4,5-THBA (lane 5), vehicle buffer control and Aβ_42_ (lane 6), and freshly prepared Aβ_42_ (lane 7). Molecular weight markers are shown in lane M.

Silver staining confirmed the presence of Aβ_42_ peptide in all of the samples but despite being a sensitive technique it did not show differences between treatment and control samples (Figure 6a). Aβ_42_ peptide was detected for all experimental samples (Figure 6a; lane 2–6) and control samples (Figure 6a; lane 1 and 7). The band intensity for monomers, dimers, and tetramers was similar in both silver staining and immunoblot analysis. However, the resolution of oligomeric species was very low, possibly due to gel smearing, which prevented quantification of individual sizes of oligomers within this range (Figure 6a).

The immunoblotting assay revealed that all of the compounds (Figure 6b; lanes 1–5) appear to be hindering fibrillogenesis when compared with the untreated control (Figure 6b; lane 6). Freshly prepared Aβ_42_ only formed monomeric and dimeric structures (<14 kDa), with no aggregation detected (Figure 6b; lane 7). In monomeric form (4.5 kDa), Aβ_42_ peptide is considered to be nontoxic [64]. However, nucleation can trigger self-assembly of these monomeric species of Aβ_42_ into oligomers, large intermediate aggregates, and eventually fibrillar aggregates [65,66]. Low molecular weight (LMW) oligomer species, distinguished by their solubility and size (>14 kDa), appear between monomeric and insoluble fibrils [67]. The level of oligomeric peptides was very high in the presence of salvianolic acid B (Figure 6b; lane 1). These results indicate the presence of high molecular weight species of oligomers and protofibrils, especially at >70 kDa (Figure 6b; lane 1). The oligomeric detection in the untreated control, on the other hand, was very low, demonstrating acceleration in formation of insoluble fibrils (Figure 6b; lane 6). All of the ThT-negative compounds were apparently capable of hindering the fibril formation but were not able to prevent complete oligomerization. Longer incubation times resulted in a slower aggregation propensity of the Aβ_42_ peptide in the presence of these selected ThT-negative compounds. These results suggest that selected compounds might act as a ligand for the β-sheet regions on the Aβ_42_, hence delaying the association of these regions for aggregation and therefore interfering with oligomerization and the fibril formation process.

## 3. Experimental Section

### 3.1. Yeast Strains

*Saccharomyces cerevisiae* BY4743 and the *AHP1* deletion (*AHP1::URA3*) mutant derived from BY4743 were used for the transformation of GFP-Aβ_42_ and GFP plasmids and flow cytometry. For the toxicity assay the *Candida glabrata* ATCC90030 strain was used.

### 3.2. Compound Library of Polyphenol Derivatives (21 Compounds)

A compound library consisting of 21 phenolic compounds and their analogues was synthesized. Syntheses of some of these compounds have been explained by Alford (2013) [68]: BA_PG31, BA_PG17, BA_PG44, BA_PG51, BA_PG87, BA_PG69, (+)-rosmarinic acid, BA_PG83, BA_PG64, BA_PG63, BA_PG65, BA_PG84, 2,3-, 2,5-, and 3,4-dihydroxybenzoic acid (DHBA), 2,4-, and 3,4-dihydroxycinnamic acid (DHCA), ferulic acid, 3,4,5-tri-hydroxybenzoic acid (gallic acid), and 2,4,5-trimethoxy cinnamic acid. The chemical structures of BA_PG compounds with significant inhibition propensity have been provided (Figure 2a).

### 3.3. Flow Cytometry Analysis of Compound-Treated Transformants

Fluorescence associated with the GFP fluorescent in yeast was measured and quantified by flow cytometry experiments. Cells for all of the following experiments were analyzed using a FACScan flow cytometer (BD FACS Canto™ II, Becton-Dickinson, Franklin Lakes, NJ, USA) and the captured files were saved as FCS3 and processed using the free trial version of WEASEL™ software (WEHI, Parkville, VIC, Australia).

The *S. cerevisiae* *AHP1* deleted strain (*Ahp1*) was transformed with plasmids encoding green fluorescent protein (GFP) and GFP fused to the C-terminus of AB_42_ (GA) using a kit (EZ-Yeast™ transformation Kit-MP, Biomedicals, Santa Ana, CA, USA). Cells were then plated on YNB without uracil for selection of transformants. Both constructs are under constitutive expression and have a *URA3* selectable marker. Prior to treatment with the compounds, transformed yeasts with the vectors were grown in selective media to exponential phase with gentle agitation (30 °C; 200 rpm). When the opacity of the culture reached OD_600nm_ of 0.8, around 150 μL of culture was transferred into a 96-well microtiter plate. All of the test compounds were dissolved in dimethyl sulfoxide (DMSO) solvents and were pre-screened for inherent fluorescence using the GFP transformants. Compounds were then added to the GA transformants at a final concentration of 50 μM. Control samples of pure DMSO were included as the negative control and growth was continued for an additional 6 h at 30 °C with shaking. Around 300 µL of the suspension was transferred into a flow cytometry tube. For measuring cell death, 3 µL of propidium iodide (PI) solution (100 μg/mL stock) was added to the flow tube to achieve the final concentration of 1 μg/mL. After addition of PI to the cells, the samples were incubated in the dark for 30 min at room temperature or for 20 min at 37 °C. Around 20,000 cells were counted in each sample and the percentage of cells exhibiting red and green fluorescence were recorded on BD FACS Canto™ II flow cytometer. The GFP green fluorescence level was measured using a FITC 530/30 filter (494/519-nm excitation/emission). Red fluorescence due to PI staining was measured with a PerCP 670LP filter (488/617-nm excitation/emission). Data were recorded and saved as FCS3 files and were analyzed by WEASEL™ (WEHI, Parkville, VIC, Australia). All compounds were analyzed in triplicate. Significant differences are indicated: * *p* < 0.05; ** *p* < 0.01; *** *p* < 0.005; **** *p* < 0.001.

### 3.4. Synthetic Aβ_42_ Peptide Preparation and Analysis

The synthetic Aβ_42_ peptide was purchased from Keck laboratories (Yale University, New Haven, CT, USA) in the form of a lyophilized powder stored at −20 °C. According to Keck laboratories, the peptide was synthesized and purified using tBOC chemistry with DCC and HOBT coupling reagents. All solvents used for the preparation of Aβ_42_ solutions were pre-filtered and centrifuged to minimize the presence of any debris that can induce aggregation of the peptide.

Peptide preparation was done according to the protocol of Ryan *et al.* [69]. Briefly, 20 mg of peptide was dissolved in 40 mL of 10% NH_4_OH to a final concentration of 0.5 mg/mL solution (w/v). The solution was then left at room temperature for 10 min and then sonicated for 5 min. This solution was then freeze dried and the peptide film (peptide pellet) was stored at −80 °C until use. Peptide concentration was determined using a quartz cuvette at A_214nm_ reading. For toxicity assays oligomeric Aβ_42_ was dissolved in 1,1,1,3,3,3-hexafuoro-2propanol (HFIP) according to the method described by Bharadwaj *et al.* [55].

### 3.5. Thioflavin T (ThT) Fluorescence Assay

Thioflavin T dye is usually used to monitor the aggregation process of Aβ peptide and quantify the extent of amyloid fibrils formation; this process has been described previously [70,71,72]. Two-millimolar stock solutions of all inhibitors were made in DMSO and stored at 4 °C or on ice. The starting ratios of Aβ to compounds were 1:1 (20 μM of each). The reactions were set up in 96-well microtiter plates in triplicate in the presence or absence of the inhibitors. Each well consisted of 2 µL of 2 mM Aβ_42_, 2 µL of 2 mM inhibitor or solvent, 20 µL 10× P_50_BS and 176 µL H_2_O to final concentration of 200 µL per well. The plates were incubated at 37 °C and samples were taken at 0, 16, and 24 h after incubation for analysis. Control wells for all solvents and the inhibitor alone were also included in the experiment to exclude any background fluorescents or aggregation related to the color of inhibitor or solvents. Aβ_42_ (20 μM) was diluted with ThT at a ratio of 1:19 by volume. The fluorescence was measured using FLUOstar OPTIMA™ multi-detection microplate reader (BMG Labtechnologies, Melbourne, VIC, Australia) with 440/480-nm excitation/ emission filters set.

### 3.6. Sample Preparation of Compounds Co-Incubated with Aβ_42_ Peptide for Gel Electrophoresis

Aβ_42_ peptide (20 μM) samples were co-incubated with the compounds (potential inhibitors) for 7 day and analyzed by gel electrophoresis. Freshly prepared Aβ_42_ was also included to indicate the effect of inhibitors and incubation time on fibril formation. All of the samples were diluted into sample buffer and boiled for 3 min and electrophoretically resolved on 4%–12% Bis-Tris pre-cast polyacrylamide gels (NuPAGE^®^ Novex^®^ LDS, Invitrogen™, Mulgrave, VIC, Australia) in MES running buffer (Cat NP0002, Invitrogen™) at 150 V for 1 h. Pre stained protein marker (SeeBlue^®^ Plus2, Cat LC5925, Invitrogen™) were run in parallel for MW comparison.

### 3.7. SDS PAGE and Silver Staining for Detection of Aβ_42_ Oligomers in the Presence of Compounds

For protein detection, a gel was stained using previously described silver staining methods [73,74]. Briefly, a gel was incubated for 30 min in 50 mL of 40% ethanol (v/v) and 10% acetic acid (w/v) and subsequently the solution was discarded and the process repeated three times. Then the gel was washed for 10 min in dH_2_O. In order to sensitize, the gel was soaked for 1 min in 0.02% Na_2_S_2_O_5_·5H_2_O and then rinsed twice with water (1 min). The gel was subsequently impregnated with silver for 20 min using silver solution (0.2% of AgNO_3_ and 0.075% HCHO). After three rinses of 20 s in water the image was developed in developing solution (0.5% HCHO, 0.0004% Na_2_S_2_O_5_·5H_2_O, 6% Na_2_CO_3_) for 10 min. Image development was stopped by adding 40% ethanol and 10% acetic acid to the gel and incubating for 10 min. Images from these gels were obtained using the VersaDoc™ imaging system (Bio-Rad Inc., Hercules, CA, USA).

### 3.8. Western-Blot for Detection of Potential Aβ_42_ Fibril Inhibitors

Aliquots of Aβ_42_ conformers (25 μM) were taken from samples of the *in situ* TEM assays after the incubation period. Fresh Aβ_42_ samples was also prepared and loaded along with 5 μL of Novex^®^ Sharp unstained protein standard (Invitrogen™). The proteins (0.5 μg) were transferred from the polyacrylamide gel to a nitrocellulose membranes using an iBlot^®^ dry system (Mulgrave, VIC, Australia). The membrane was then blocked for 1 h in 5% non-fat dry skim milk in PBS solution and washed three times with PBS. Primary antibody WO2 [75] (University of Melbourne, VIC, Australia), attached to the N-terminal of Aβ, was used for detection. This antibody was diluted in PBS/Casein solution (1/200; 0.5% skim milk in PBS) prior to use. Incubation of the blot in primary antibody for 2 h continued and was followed by three washes with PBS. Secondary antibody (anti-mouse) was diluted to 1/1000 and incubated on the membrane for 1 h. After washing with PBS, the blot membrane was exposed to ECL chemiluminescent western blotting substrate (ThermoFisher Scientific) for 2 min and allowed to develop. Images were captured using the VersaDoc™ imaging system (Bio-Rad Inc.). The immunoreactive bands were later quantified using Quantity One 1-D analysis software (Bio Rad, Australia; version 4.6.8).

### 3.9. Transmission Electron Microscope (TEM) Imaging

Carbon-coated 300-mesh copper grids were glow discharged in nitrogen to render the carbon film hydrophilic. Samples were gently agitated before transferring a 4 μL aliquot onto the grids. After 30 s adsorption time, the excess sample was drawn off using a Whatman^®^ 541 filter paper. The grids were then subsequently stained with 2% w/v potassium phosphotungstate (pH 7.2) for 10 s. The grids were then air dried before examination. The samples were examined using a Tecnai™ 12 Transmission Electron Microscope (FEI, Eindhoven, Netherlands) at an operating voltage of 120 KV. Images were recorded using a Megaview III CCD camera and AnalySIS camera control software (Olympus Australia, Macquarie Park, NSW, Australia).

#### Sample Preparation for TEM

Freshly prepared aliquots of Aβ_42_ peptide were used for the treatment of the chemicals. The samples were set up in screw cap Eppendorf tubes containing 20 μM Aβ_42_ peptide, 20 μM of the inhibitors, and 20 μL of a 10× phosphate buffer stock that gave a final concentration of 50 mM KH_2_PO_4_/300 mM NaCl pH 7.5 The samples were made up to the final volume of 200 μL by adding H_2_O. The control sample contained equal amounts of peptide and inhibitor solvents. All of the samples were incubated at 37 °C for 6 day for TEM analysis.

### 3.10. Statistical Analysis

All samples were analyzed in triplicate and experiments were repeated to ensure their reproducibility. Graphs were made using Microsoft Office Excel 2010 software and data were analyzed using PRISM 5 version 5.04 (GraphPad Software, Inc., La Jolla, CA, USA). All data have been presented as mean ± SEM. Significant differences were compared using unpaired Student’s *t-*test and one-way ANOVA with either Bonferroni’s or Tukey’s *post hoc* analysis. Significant differences are indicated by asterisks (*) and the corresponding *p*-values for the data are given in the text. A *p*-value of <0.05 was deemed significant.

## 4. Conclusions

The inhibition of Aβ_42_ aggregation or disaggregation of the preformed oligomers by phenolic compounds that form part of the natural diet show promising evidence that these compounds have anti-amyloidogenic and AD properties [34,76,77,78]. Herewith, using a yeast model of AD, we have identified the phenolic compounds that reduced ThT-associated fluorescence and were also capable of reducing the green fluorescence associated with the GFP-Aβ_42_. These compounds also provided protection against toxicity induced by the HFIP-pretreated Aβ_42_ peptide. It is known that the protective effect of many phenolic compounds on the aggregation and neurotoxicity effect of Aβ_42_ peptide is not their only affect [79,80] but they are also able to reduce the inflammation and oxidative stress exacerbated by the accumulation of Aβ_42_ peptide. For example, caffeic acids, if present in appreciable concentrations, are known to possess high antioxidant activity due to the presence of 3,4-dihydroxyphenyl moieties and electron-donating groups [81].

All of the known polyphenols that are inhibitors of Aβ_42_ aggregation are composed of at least two phenolic rings with a minimum of two hydroxyl groups on the aromatic rings, that enables them to strongly interact with peptide residues in β-sheet regions of oligomers [82]. Our results support the view that certain DHBs destabilize and dissociate amyloid oligomer structure reversibly into monomeric amyloids [46]. For example, the relative placement of hydroxyl groups has a profound effect on chemical activity/redox activity and other interactions. The phenolic hydroxyl groups are capable of neutralizing free radicals, forming aryloxy radicals in the process [83]. Chemically, vicinal hydroxyl groups on aromatic rings can be oxidized to *ortho* quinones thereby exerting antioxidant effects [84] unlike the 1,3 dihydroxy isomer that can only exert electrophilic activity [85]. The results here revealed that the positions of the hydroxyl moieties on the aromatic rings are the major determinant of their potency and anti-aggregation rather than the number of hydroxyl groups as illustrated by the data for 2,5-DHBA shown in Figure 1 and Figure 5

The benzofuran moiety has also been reported as a potential inhibitor of Aβ_42_ fibril formation [39,40,86]. The inhibitory activity is reportedly due to its ability to bind to Aβ_42_ [86]. This suggests that specific recognition sites for any potential inhibitors on the Aβ_42_ peptide, such as the amyloid C-10 to C-21 hydrophobic region, bind and interfere with the aggregation and fibril formation capacity of the peptide. Our ThT analyses for the benzo[b]furan scaffold compounds indicated that their inhibitory effect is temporary. Therefore, further investigation into their underlying mechanism of interactions with the Aβ_42_ peptide is required to elucidate their potential application. Although selected molecules show promising results in the prevention or perhaps the treatment of cognitive deficits, as well as the inhibition of the development of AD, their bioavailability and toxicology in human subjects requires further investigation prior to their clinical application.
